# Latent Cluster Analysis of ALS Phenotypes Identifies Prognostically Differing Groups

**DOI:** 10.1371/journal.pone.0007107

**Published:** 2009-09-22

**Authors:** Jeban Ganesalingam, Daniel Stahl, Lokesh Wijesekera, Clare Galtrey, Christopher E. Shaw, P. Nigel Leigh, Ammar Al-Chalabi

**Affiliations:** Department of Clinical Neuroscience, MRC Centre for Neurodegeneration Research, King's College London, Institute of Psychiatry, London, United Kingdom; University of Cambridge, United Kingdom

## Abstract

**Background:**

Amyotrophic lateral sclerosis (ALS) is a degenerative disease predominantly affecting motor neurons and manifesting as several different phenotypes. Whether these phenotypes correspond to different underlying disease processes is unknown. We used latent cluster analysis to identify groupings of clinical variables in an objective and unbiased way to improve phenotyping for clinical and research purposes.

**Methods:**

Latent class cluster analysis was applied to a large database consisting of 1467 records of people with ALS, using discrete variables which can be readily determined at the first clinic appointment. The model was tested for clinical relevance by survival analysis of the phenotypic groupings using the Kaplan-Meier method.

**Results:**

The best model generated five distinct phenotypic classes that strongly predicted survival (p<0.0001). Eight variables were used for the latent class analysis, but a good estimate of the classification could be obtained using just two variables: site of first symptoms (bulbar or limb) and time from symptom onset to diagnosis (p<0.00001).

**Conclusion:**

The five phenotypic classes identified using latent cluster analysis can predict prognosis. They could be used to stratify patients recruited into clinical trials and generating more homogeneous disease groups for genetic, proteomic and risk factor research.

## Introduction

Amyotrophic lateral sclerosis (ALS) is a degenerative disease of motor neurons resulting in progressive paralysis and death from respiratory failure within three to five years [1]. The cause of sporadic ALS (SALS) is unknown but genetic analyses show disease heterogeneity for familial cases, and this is likely for SALS as well. This is a problem for research into risk factors as the effective sample size is reduced with a concomitant reduction in power. Similarly, the search for biomarkers is hampered if there are several underlying disease processes with similar clinical phenotypes [2], [3]. Furthermore, the effectiveness of a drug may be masked if a clinical trial does not take into account heterogeneity in survival. This is particularly important if the different disease mechanisms respond to different therapies.

ALS has been classified using various systems, the best known of which is based on predominant site of symptom onset and the predominance of upper and lower motor neuron signs at presentation: progressive bulbar palsy, pseudobulbar palsy, progressive muscular atrophy, primary lateral sclerosis and amyotrophic lateral sclerosis. The El Escorial criteria and its descendants confer diagnostic certainty based on the regional distribution of upper and lower motor neuron signs that distinguishes them from other motor neuron disorders.[4]–[7]. These classification systems depend on agreement between clinicians who specialise in ALS to recognize underlying disease patterns, and as such are subjective. We sought to explore whether clinical or demographic variables available to a clinician tended to occur in a predictable pattern that might be apparent to unbiased statistical analysis and could therefore be used to dissect out underlying disease types in an objective way. We restricted the variables to those available at a first visit as these would be the most useful for clinical prognostication and in clinical trials.

## Methods

### Patients

A tertiary referral centre clinical database containing information on 1467 people with motor neuron disease was analysed. All patients included were diagnosed as having ALS or an ALS variant by at least two consultant neurologists after full investigation to exclude other conditions between 1993 and 2007. The study was approved by the Institutional Research Ethics Committee.

### Clinical variables

The variables selected were: age of onset of weakness, sex, ethnicity, family history of ALS in a first degree relative, site of onset of first symptoms, diagnostic delay (interval between first symptoms of weakness and diagnosis), physician-classified phenotypic group and the number of functional regions affected. Because vital capacity was not available for a large proportion of patients, this was not included in the analysis. Functional regions were defined as bulbar, upper limb, lower limb and respiratory. Symptoms or signs defined involvement. Respiratory involvement was defined by the presence of orthopnoea, breathlessness on minimal exertion, or forced vital capacity or sniff nasal inspiratory pressure less than 70% of predicted. The physician-classified phenotypic groups were progressive muscular atrophy (lower motor neuron signs only), amyotrophic lateral sclerosis (upper and lower motor neuron signs fulfilling the El Escorial criteria for possible, probable or definite ALS), primary lateral sclerosis, flail arm syndrome (brachial amyotrophic diplegia as defined previously [8], [9] and flail leg syndrome (pseudopolyneuritic variant of ALS) [10].

### Statistical methods

We used latent class cluster analysis (LCCA) to explain associations between observed manifest indicator variables (clinical observations) through hypothesized underlying unobserved latent variables. LCCA is a model based cluster analysis method used to identify subtypes of related cases (latent classes) from categorical, ordinal and continuous multivariate data [11]–[14]. The method assumes *k* latent groups or latent classes underlying the data set and that each case belongs to only one group. The number of classes and their sizes are not known *a priori*. LCCA uses maximum likelihood estimation methods to minimize association among the responses across multiple observed variables. It recognizes that there is some degree of uncertainty in the classification by assigning each case a posterior probability of belonging to each class.

To estimate the number of classes underlying the sample, we compared the fit of models with increasing numbers of classes using three different methods. Firstly values of the Akaike information criteria (AIC) and Bayesian information criteria (BIC) were used to estimate the optimal number of classes. Lower AIC and BIC values suggest better fitting models. Secondly, we used the model entropy, an overall measure of how well a model predicts class membership, which ranges from 0 (no predictive power) to 1 (perfect prediction) [15]. Thirdly, we used the mean posterior probability of a case belonging to each class. A good fitting model would have high individual probabilities for each case belonging to just one class since this is one of the underlying assumptions. A case was assigned to the latent class that corresponded to the highest (modal) posterior conditional response probability across the indicator variables. LCCA statistical analyses were carried out in MPlus 5.1 [14].

To characterize the latent classes and to identify the clinical variables that best described class membership we used discriminant function analysis (DFA) and multinomial regression [16]. Categorical variables were included as dummy coded variables in the DFA. DFA determines *n* functions, (where *n* is the smaller of the number of groups-1 or the number of variables), in a way that the first function provides the most overall discrimination between groups, the second provides second most, and so on. DFA allows visualization of how the two functions discriminate between groups by plotting the individual scores for the two first discriminant functions.

DFA assumes continuous and normally distributed data. Although DFA is known to perform reasonably well when using dummy coded variables [17], [18], we used a multinomial regression with robust standard errors to confirm the conclusions derived from the DFA. Multinomial regression is an extension of logistic regression to categorical dependent variables with more than two outcomes. Multinomial regression allows the use of both categorical and continuous independent variables and the predictors do not have to be normally distributed, linearly related, or of equal variance within each group [16].

To validate the model clinically, we performed a Kaplan-Meier survival analysis to test if the classes had prognostic value, since survival was not a variable used in the cluster analysis. Discriminant analysis and Kaplan Meier analysis were performed in SPSS v15.0 (SPSS Inc) and multinomial regression in STATA 10.1 (STATA Inc.).

## Results

### Latent class model selection

A five class model gave the best fit (Table 1), with the lowest AIC and BIC values (Table S1.) Using six or more classes did not result in convergence to any underlying model even after increasing the number of iterations and using different starting values. Further evidence that the five-class solution was the most parsimonious was that most cases could easily be assigned to just one class, with high mean posterior probabilities of class membership ranging from 86.1 to 100% (Table 1). Furthermore, the entropy of the five class model was 0.842, a good overall certainty in classification.

**Table 1:** Latent class membership based on the estimated posterior probability.

Class	Based on estimated posterior probabilities	Based on most likely class membership	Class 1	Class 2	Class 3	Class 4	Class 5
1	728.4 (49.7%)	**763 (52%)**	**86.1%**	12.1%	0.0%	1.8%	0.0%
2	558.8 (38.1%)	**527 (35.9%)**	11.5%	**88.1%**	0.0%	0.4%	0.0%
3	4 (0.3%)	**4 (0.3%)**	0.0%	0.0%	**100.0%**	0.0%	0.0%
4	133.6 (9.1%)	**130 (8.9%)**	8.4%	1.7%	0.0%	**89.7%**	0.2%
5	42.2 (2.9%)	**43 (2.9%)**	0.0%	0.0%	0.0%	2.9%	**97.1%**

The first column shows the membership based on the mean posterior probability for each class. The second column shows the number of subjects (%) classified in a given class based on their most likely average latent class membership (row) by latent class (column). For example: The estimated average posterior probability of belonging to Class 1 is 49.7% corresponding to an estimated sample size of 728.8 subjects in this class. 52% of the subjects were classified into Class 1 based on their highest posterior probability. Their average posterior probability for membership of Class 1 was 86.1%, while their probability of belonging to Classes 2, 3, 4 or 5 was 12.1%, 0%, 1.8% and 0% respectively.

### Characteristics of a five-class solution

**Figure 1:**
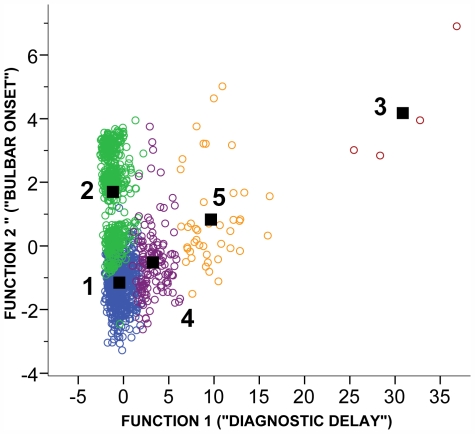
A plot of the location of each case on the first two axes of the discriminant function analysis. Circles have been coloured according to assigned class. (Blue - class 1, Green - class 2, Red – class 3, Purple – class 4, Orange – class 5). The black square represents the centroid for each group distribution. Discriminant function 1 corresponds mainly to time to diagnosis from symptom onset (diagnostic delay), while discriminant function 2 corresponds mainly to bulbar onset (higher values) with some contribution from clinical phenotype and age of onset (see Table 3).

The discriminant function analysis revealed two main functions that explained 98.9% of the total variance (Table S2). The first function mainly corresponded to delay between first symptoms and diagnosis, while the second function mainly corresponded to site of onset of first symptoms (bulbar or not) and to a lesser extent, clinical phenotype and age of onset. Figure 1 shows a plot of the individuals of each group on the first two discriminant dimensions. The five latent classes are clearly separated by the two functions and a jack-knife cross-validation reveals a very high correct classification rate of almost 90% (Table S3). The results were confirmed by a multinomial regression with classes 1, 2, 3 and 4 as the dependent variables. Stepwise model selection revealed that bulbar onset and diagnostic delay were the best predictors of class membership (Table S4). 86.1% of the cases were correctly classified using only those two variables (Table 2), suggesting that two variables alone, diagnostic delay and site of onset of first symptoms, were quite effective at predicting group membership.

**Table 2:** Prediction matrix based on multinomial regression.

Class	1	2	4	5	Total
**1**	**748 (98%)**	1 (0.1%)	8 (1%)	6 (0.8%)	763
**2**	170 (32.3%)	**351 (66.6%)**	3 (0.6%)	3 (0.6%)	527
**4**	11 (8.5%)	1 (0.8%)	**117 (90%)**	1 (0.8%)	130
**5**	0 (0%)	0 (0%)	0 (0%)	**43 (100%)**	43
**Total**	929	353	128	53	1463

Observed classes are in rows, predicted in columns. Overall correct classification rate was 86.1%. Class 3 was omitted because of small sample size. Classes 1, 2 and 4 show a very high predictive probability using diagnostic delay and bulbar onset. However the predicted Class 2 appears to include cases that appeared in Class 1. This may require the use of other variables not included in this study to better distinguish between Classes 1 and 2.

Class 1 was characterized by non-bulbar onset and a very short diagnostic delay of as little as 2 months (Figure 1 and Table 3). Class 2 was characterized by a similar, but slightly shorter diagnostic delay, the major difference with class 1 being a higher predominance of bulbar onset. The two classes also differed in clinical phenotype assigned by the neurologist. Class 2 consisted almost entirely of those with ALS, while class 1 also included those with progressive muscular atrophy, flail arm and flail leg phenotypes. Furthermore those in Class 1 tended to be younger than those in Class 2. Interestingly, the only class in which the normal male excess was reversed was Class 2.

**Table 3:** Characteristics of the subjects within each latent class.

Variable	Class	1	2	3	4	5	Total
**Gender**	Male	596 (78.1%)	200 (38%)	3 (75%)	72 (55.4%)	30 (69.8%)	901 (61.4%)
	Female	167 (21.9%)	327 (62%)	1 (25%)	58 (44.6%)	13 (30.2%)	566 (38.6%)
**Ethnicity**	White	714 (93.6%)	512 (97.2%)	4 (100%)	117 (90.7%)	38 (88.4%)	1385(94.5%)
	Black	16 (2.1%)	9 (1.7%)	0 (0%)	4 (3.1%)	3 (7%)	32 (2.2%)
	Other	33 (4.3%)	6 (1.1%)	0 (0%)	8 (6.2%)	2 (4.7%)	49 (3.3%)
**Age at onset**	(years)	54.4 (12.3)	61.3 (11.2)	45.3 (14.6)	55.9 (12.2)	52.6 (11.8)	56.9 (12.3)
**Family history**	Yes	45 (5.9%)	34 (6.5%)	2 (50%)	5 (3.8%)	1 (2.3%)	87 (5.9%)
	No	718 (94.1%)	493 (93.5%)	2 (50%)	125 (96.2%)	42 (97.7%)	1380(94.1%)
**Number of regions**	(number)	4.2 (0.8)	4.7 (0.81)	4.3 (0.96)	3.9 (1.14)	4.1 (1.06)	4.4 (0.89)
**Diagnostic delay**	(months)	13.1 (7.7)	10.9 (6.6)	280.3 (43.5)	44.9 (10.6)	99.8 (21.1)	18.4 (23.5)
**Bulbar onset**	Bulbar	1 (0.1%)	354 (67.2%)	1 (25%)	6 (4.6%)	8 (18.6%)	370 (25.2%)
	Non-bulbar	762 (99.9%)	173 (32.8%)	3 (75%)	124 (95.4%)	35 (81.4%)	1097(74.8%)
**Phenotype**	PMA	42 (5.5%)	1 (0.2%)	0 (0%)	11 (8.5%)	2 (4.7%)	56 (3.8%)
	Flail Arm	122 (16%)	0 (0%)	0 (0%)	24 (18.5%)	6 (14%)	152 (10.4%)
	Flail Leg	50 (6.6%)	0 (0%)	0 (0%)	28 (21.5%)	2 (4.7%)	80 (5.5%)
	ALS	536 (70.2%)	519 (98.5%)	2 (50%)	40 (30.8%)	18 (41.9%)	1115(76%)
	PLS	13 (1.7%)	7 (1.3%)	2 (50%)	27 (20.8%)	15 (34.9%)	64 (4.4%)

Values show means (SD) and counts (%). This table demonstrates how variables that we have traditionally used to sub-divide and study ALS associate with the groups generated in this study. While not showing significant differences, we can see trends suggesting that age of onset is associated with speed of progression. There are some sex ratio differences, particularly between Classes 1 and 2. However, strikingly, there is no clear split with the traditionally used phenotypes.

The four members of Class 3 were clearly separated from the members of the other classes by an extremely long diagnostic delay of at least 234 months. Classes 4 and 5 were also mainly separated from each other and from the other two classes by diagnostic delay, which ranged from 26–70 months in class 4 and from 74 to 158 months in class 5 (Figure S1). Most members of class 4 had non-bulbar onset, while almost 20% of the members of class 5 did have bulbar onset. Members of class 4 had a higher proportion of those with a flail leg phenotype than other classes. Family history, ethnicity and number of symptomatic regions were similar across all classes.

### Validation analysis

**Figure 2:**
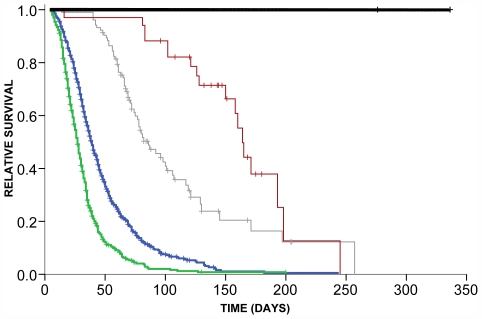
Kaplan Meier Survival curve for the five different classes. Each line is coloured according to assigned class (Blue - class 1, Green – class 2, Black – class 3, Grey – class 4, Red – class 5). Crosses represent cases that are censored. Survival is significantly different between any pair of classes. Class 3 consists of only three subjects.

A Kaplan-Meier analysis of 1311 cases showed good separation for survival curves of each class (Logrank test chi^2^(3) = 340.2, p<0.0001, Figure 2). Class 3 was excluded from the statistical analysis because of small sample size. Survival of each class was significantly different from each other group and 95% confidence intervals did not overlap between the four classes showing that the latent class groupings have prognostic value (Table 4). Median survival was shortest for patients of Class 2 followed by Classes 1, 4 and 5. Survival time was longest for the three cases of Class 3 with known survival.

**Table 4:** Median survival.

Class	N	Median in months (95% C.I.)	Logrank test for pairwise comparisons		
			**Class 2**	**Class 4**	**Class 5**
**1**	686	39 (36.6–41.4)	χ^2^ = 100.7, p<0.001	χ^2^ = 98.5, p<0.001	χ^2^ = 84.0, p<0.001
**2**	484	27 (25.2–28.7)		χ^2^ = 191.1, p<0.001	χ^2^ = 99.5, p<0.001
**3**	3	>76, >36, >276			
**4**	104	86 (69.7–102.3)			χ^2^ = 14.2, p<0.001
**5**	34	164 (154.3–173.5)			

Median survival is shown in months with 95% confidence intervals for Classes 1, 2, 4, and 5 and the results of pairwise comparisons of the survival curve using a log rank test. Because for Class 3 the survival times were available for only three patients, the individual censored survival times are shown. Note that the confidence intervals do not overlap between the groups.

## Discussion

We have applied a latent class cluster analysis to a database of over 1467 people with ALS to identify clinical sub-groups that have prognostic value. We used eight clinical variables that can be easily assessed at the first visit to generate a five class model. Two variables alone, site of first symptoms (bulbar or limb) and time to diagnosis from first symptoms were sufficient to classify most people accurately. Discriminant function and multinomial regression analyses allowed us to convert this mathematical construction into a clinically useful tool in which the two major contributors to the classification were bulbar or limb onset and diagnostic delay. However, classification additionally depended on the distribution of phenotypes and to a lesser extent age of onset. Cluster analysis will draw out clusters based on factors that have the largest impact on classification. The exclusion of age from the simplified, two-factor model does not mean that age is not relevant, but that it can be subsumed into the information available from site of onset and disease duration. Also closer inspection of Table 3 reveals gender ratio differences between the classes, particularly between Classes 1 and 2. As a concurrent validation of the LCCA classification we used survival duration as an external criterion of clinical relevance. The five classes had significantly differing, non-overlapping survival durations, which suggest that the classification is clinically relevant. The prognostic value of this model suggests that we have found groups that could potentially correlate with differences in pathological mechanisms [19], [20]. It is of interest that over 1200 of the 1467 patients are grouped into just two classes. This suggests that ALS as a whole is more homogenous than is often reported.

Latent class clustering is a model-based technique that assumes that data are generated by a mixture of probability distributions [13]. This makes it different from classical cluster analysis, such as *k*-means clustering, which is based on a statistical measure of distances between observations. Associations among the observed (manifest) variables, in this case clinical observations at the first clinic visit, are explained through hypothesized unobserved (latent) categorical variables. Manifest variables are therefore assumed to be independent within each latent class. LCCA can be seen as a categorical analogue of factor analysis. However, factor analysis analyzes the structure of manifest variables, whereas LCCA is more concerned with the structures of cases.

LCCA has several additional advantages over traditional cluster analysis methods. Firstly, classification based on posterior probability allows assessment of the quality of classification. Secondly, it can deal with a mix of nominal, ordinal, count or continuous variables, any of which may contain missing values. Thirdly, because LCCA is scale independent, data do not need to be standardized. Fourthly, because LCCA is based on a statistical model, statistics such as information criteria can be used to objectively determine the number of classes in the data. LCCA is also objective because it does not use a clustering algorithm and so the choice of clustering algorithm and its effects on results is not an issue. We are therefore confident that four of the five classes we have identified represent an objective classification of ALS phenotypes. Class 3 consisted of only four cases and therefore should be treated with caution, and larger numbers would be useful for confirmation. Finally, LCCA is a type of latent variable methodology [21], and therefore allows flexible modelling such as including covariates in the model or lowering the restrictions of local independence.

There are limitations to this study. The categorical nature of five of the seven variables meant that reduction of the sample size to replicate the latent class structure and cross-validate the results was not possible. Further studies are therefore needed to confirm the existence and characteristics of the five distinct classes.

There are many current classification systems for ALS. The oldest is based on the distinction between upper and lower motor neuron involvement and site of predominant disease burden, with categories progressive bulbar palsy, pseudobulbar palsy, progressive muscular atrophy, primary lateral sclerosis and amyotrophic lateral sclerosis. In 1999, a classification was proposed based on the underlying causative mechanisms and acknowledgement of different phenotypes where cause was unknown [4]. Subsequently, the El Escorial criteria were established for research purposes, and primarily to assist in recruitment for clinical trials [22]. These have been superseded by the Airlie House criteria [23] and may be superseded again [6]. Unfortunately there are several well recognised problems with the El Escorial criteria and their revisions. Up to 40% of patients may be excluded from research despite there being little clinical doubt about the diagnosis. Patients with bulbar onset of symptoms, which is associated with a reduced life expectancy, may never fulfil the El Escorial criteria. People with atypical presentations can be difficult to classify and are therefore excluded. Other limitations include the focus on extent of the disease rather than burden of the disease, the lack of discrimination between bulbar and spinal symptom onset and the poor correlation with prognosis, although it is acknowledged that this was not the purpose of the classification [5], [7]. More recent classifications have called for detailed phenotypic groupings of lower motor neuron [10], [24] or upper motor neuron [25] syndromes, or for a distinction between proximal, symmetrical disease and distal, asymmetrical disease [26]. An ideal classification would be one that reflects homogeneity of an underlying disease mechanism within each group or has clinical relevance, for example in predicting prognosis. Either of these properties would enable a classification to be truly useful in clinical trials because patients could be stratified by prognostic group and disease process, improving power. The classification we propose has at least the property of being prognostically useful and may reflect underlying disease groups each with differing mechanisms. The main difficulty in generalising this system is that the delay between symptom onset and diagnosis will depend to a large extent on the local health care system and other local variables. Since this is the most important classification variable, the equation for class membership would need to be calibrated for each clinic. This is not insurmountable however, if it is recognised that the diagnostic delay is simply a marker of the rate of disease progression [1]. There are other equivalent markers that will be invariant between geographical sites, such as the time to develop symptoms in a second functional region, and these could be used to generate an equivalent classification.

The variables in the model include a classification of the disease type based on the pattern reported by the examining clinician. This consists of the traditional phenotypic categories but further subdivides those with progressive muscular atrophy into flail arm or flail leg phenotypes for those with proximal symmetrical disease. Although such a classification has prognostic value, it does not explain the classes defined by this latent class cluster analysis (Table S5). The key question facing researchers is whether we should be ‘lumpers’ or ‘splitters’ in classifying ALS [27], [28]. This needs to be resolved to make further progress in genetics, biomarker and drug discovery. The persistence in lumping patients in the clinical design of drug trials maybe one of the main reasons for the lack of success in finding disease modifying therapies [29].

While we do not suggest this is the final model to be applied in trials, biomarker discovery and genetic studies, the challenge is to characterise further the classes we have identified. Genome-wide association studies and protein biomarkers may help in delineating underlying biological differences between the classes. Further clinical variables may also assist in fine-tuning the classification. For example, we know that up to 50% of patients demonstrate minimal cognitive impairment with a significant proportion having FTLD [30]. It would also be interesting to know if this model can predict secondary end-points such as the time to use of non-invasive ventilation, or time to insertion of gastrostomy.

In summary, sub-groups defined by latent class cluster analysis show statistically significant differences in survival and the classification system might therefore be used to stratify patients in clinical trials, and to generate more homogeneous groups for genetic, proteomic and other risk factor research.

## Supporting Information

Table S1 Comparison of latent class analysis models. Information criteria (AIC and BIC) and Entropy evaluate the quality of different latent class solutions. Smaller AIC and BIC values suggest a better fitting model. A five class model (bold) has the best fit based on information criteria.
(0.03 MB DOC)

Table S2 Results of discriminant function analysis. The percentage of explained variance is the percentage of discriminating power for the model associated with a given discriminant function. The canonical correlation is a measure of the association between the groups formed by the tested variable and a given discriminant function. Figures given for each variable are the factor structure coefficients, which are the pooled within-groups correlations between the variables in the model, and the standardized canonical discriminant functions. Correlations >0.5 are printed in bold and are considered the variables best associated with a given function.
(0.05 MB DOC)

Table S3 Leave-one-out cross-classification (Jacknife). Rows are the observed classes and the columns are the predicted classes of the cases. Each subject has been classified using a discriminant function analysis based on all cases except the given case. 90.1% of the original grouped cases and 89.9% of the cross-validated groups were correctly classified.
(0.03 MB DOC)

Table S4 Result of multinomial regression analysis. Class 1 is the reference group. The standard interpretation of a multinomial logit model is that for one unit change (or change from one category to another) of the independent variable, the logit of the outcome relative to the reference group (Class 1) is expected to change by the respective parameter estimate. A positive regression coefficient implies that the probability of belonging to the reference group (Class 1) decreases. Class 3 was not included because of small sample size. The overall model Wald chi2 was 861.8, P<0.00001.
(0.04 MB DOC)

Table S5 Binned adjusted standardised residuals of a chi2 cross-tabulation analysis between phenotype and class. Arrows show the direction of deviation. Adjusted standardised residuals outside the range −2.5 and +2.5 indicate significant departure from independence. Adjusted standardised residuals <−8 or >+8 are considered as extreme departures from independence. Positive adjusted residuals in a cell correspond to larger numbers of cases than expected by chance, negative residuals smaller numbers. Class 3 was excluded from the statistical analysis because of the small sample size.
(0.03 MB DOC)

Figure S1 Histogram showing the distribution of diagnostic delay for cases within each class, separately for patients with and without bulbar onset. The frequency (Y-Axis) is shown on a logarithmic scale.
(0.27 MB TIF)
