# Supplementary material for: Latent Cluster Analysis of ALS Phenotypes Identifies Prognostically Differing Groups
Source: PLoS One. 2009 Sep 22;4(9):e7107. doi: 10.1371/journal.pone.0007107 (PMC2741575; doi:10.1371/journal.pone.0007107)
Supplement: Table S1 — Comparison of latent class analysis models. Information criteria (AIC and BIC) and Entropy evaluate the quality of different latent class solutions. Smaller AIC and BIC values suggest a better fitting model. A five class model (bold) has the best fit based on information criteria. (0.03 MB DOC) [file pone.0007107.s001.doc]

|  | *1 class* | *2 classes* | *3 classes* | *4 classes* | **5 classes** |
| --- | --- | --- | --- | --- | --- |
| AIC | 35505 | 34658 | 33986 | 33374 | 33018 |
| BIC | 35595 | 34833 | 34245 | 33718 | 33446 |
| Entropy | 1 | 0.989 | 0.989 | 0.835 | 0.842 |
| **Class size** | 1467 | 1420:47 | 1386:4:77 | 4:71:725:667 | 763:527:4:130:43 |
