# Supplementary material for: Latent Cluster Analysis of ALS Phenotypes Identifies Prognostically Differing Groups
Source: PLoS One. 2009 Sep 22;4(9):e7107. doi: 10.1371/journal.pone.0007107 (PMC2741575; doi:10.1371/journal.pone.0007107)
Supplement: Table S2 — Results of discriminant function analysis. The percentage of explained variance is the percentage of discriminating power for the model associated with a given discriminant function. The canonical correlation is a measure of the association between the groups formed by the tested variable and a given discriminant function. Figures given for each variable are the factor structure coefficients, which are the pooled within-groups correlations between the variables in the model, and the standardized canonical discriminant functions. Correlations >0.5 are printed in bold and are considered the variables best associated with a given function. (0.05 MB DOC) [file pone.0007107.s002.doc]

| **Result** | **Category** | **DF 1** | **DF 2** | **DF 3** | **DF 4** |
| --- | --- | --- | --- | --- | --- |
| Eigenvalue |  | 7.0 | 1.4 | 0.1 | <0.01 |
| % explained variance |  | 82.1 | 16.8 | 0.9 | 0.2 |
| Canonical correlation |  | 0.94 | 0.77 | 0.27 | 0.11 |
| **Variable** |  |  |  |  |  |
| Diagnostic delay |  | **0.99** | 0.11 | -0.02 | 0.01 |
| Bulbar onset | Yes | -0.08 | **0.86** | -0.25 | -0.02 |
| Number of symptomatic regions |  | -0.05 | 0.26 | 0.21 | -0.08 |
| Phenotype | PMA | 0.02 | -0.12 | -0.14 | 0.07 |
|  | Flail arm | 0.03 | -0.22 | -0.08 | -0.06 |
|  | Flail leg | 0.04 | -0.15 | **-0.57** | **0.55** |
|  | ALS | -0.12 | 0.33 | **0.75** | -0.08 |
|  | PLS | 0.15 | -0.01 | **-0.55** | -0.46 |
| Age of onset |  | -0.04 | 0.21 | -0.23 | 0.16 |
| Ethnicity | White | -0.02 | 0.07 | 0.12 | 0.24 |
|  | Black | 0.02 | -0.01 | -0.07 | -0.36 |
|  | Other ethnicity* | 0.01 | -0.08 | -0.10 | -0.02 |
| Family history | Yes | 0.01 | 0.02 | 0.24 | 0.61 |
