# Supplementary material for: Latent Cluster Analysis of ALS Phenotypes Identifies Prognostically Differing Groups
Source: PLoS One. 2009 Sep 22;4(9):e7107. doi: 10.1371/journal.pone.0007107 (PMC2741575; doi:10.1371/journal.pone.0007107)
Supplement: Table S3 — Leave-one-out cross-classification (Jacknife). Rows are the observed classes and the columns are the predicted classes of the cases. Each subject has been classified using a discriminant function analysis based on all cases except the given case. 90.1% of the original grouped cases and 89.9% of the cross-validated groups were correctly classified. (0.03 MB DOC) [file pone.0007107.s003.doc]

| **Class** | **1** | **2** | **3** | **4** | **5** | **Total** |
| --- | --- | --- | --- | --- | --- | --- |
| **1** | 681 (95.4%) | 1 (0.1%) | 0 (0%) | 32 (4.5%) | 0 (0%) | 714 |
| **2** | 169 (32.3%) | 351 (67%) | 0 (0%) | 4 (0.8%) | 0 (0%) | 524 |
| **3** | 0 (0%) | 0 (0%) | 4 (100%) | 0 (0%) | 0 (0%) | 4 |
| **4** | 0 (0%) | 0 (0%) | 0 (0%) | 128 (100%) | 0 (0%) | 128 |
| **5** | 0 (0%) | 0 (0%) | 0 (0%) | 1 (2.3%) | 42 (97.7%) | 43 |
