# Supplementary material for: Latent Cluster Analysis of ALS Phenotypes Identifies Prognostically Differing Groups
Source: PLoS One. 2009 Sep 22;4(9):e7107. doi: 10.1371/journal.pone.0007107 (PMC2741575; doi:10.1371/journal.pone.0007107)
Supplement: Table S4 — Result of multinomial regression analysis. Class 1 is the reference group. The standard interpretation of a multinomial logit model is that for one unit change (or change from one category to another) of the independent variable, the logit of the outcome relative to the reference group (Class 1) is expected to change by the respective parameter estimate. A positive regression coefficient implies that the probability of belonging to the reference group (Class 1) decreases. Class 3 was not included because of small sample size. The overall model Wald chi2 was 861.8, P<0.00001. (0.04 MB DOC) [file pone.0007107.s004.doc]

|  | ***Coefficient (95% C.I.)*** | ***robust SE*** | ***z*** | ***P*** |
| --- | --- | --- | --- | --- |
| **Class 2** |  |  |  |  |
| Bulbar onset = yes | 7.34 (5.37-9.31) | 1.00 | 7.31 | <0.0001 |
| Diagnostic delay | -0.04 (-0.07-(-0.02)) | 0.01 | - 3.43 | <0.001 |
| Constant | -0.98 (-1.3-(-0.66)) | 0.16 |  |  |
| **Class 4** |  |  |  |  |
| Bulbar onset = yes | 0.77 (-5.63-7.17) | 3.27 | 0.24 | 0.81 |
| Diagnostic delay | 0.59 (0.46-0.73) | 0.07 | 8.78 | <0.0001 |
| Constant | -19.24 (-23.64-(-14.83)) | 2.25 |  |  |
| **Class 5** |  |  |  |  |
| Bulbar onset = yes | 98.12 (85.09-111.15) | 6.65 | 14.76 | <0.0001 |
| Diagnostic delay | 7.97 (7.26-8.68) | 0.36 | 22.13 | <0.0001 |
| Constant | -550.31 (-600.44-(-500.19)) | 25.57 |  |  |
