# Supplementary material for: Latent Cluster Analysis of ALS Phenotypes Identifies Prognostically Differing Groups
Source: PLoS One. 2009 Sep 22;4(9):e7107. doi: 10.1371/journal.pone.0007107 (PMC2741575; doi:10.1371/journal.pone.0007107)
Supplement: Table S5 — Binned adjusted standardised residuals of a chi2 cross-tabulation analysis between phenotype and class. Arrows show the direction of deviation. Adjusted standardised residuals outside the range −2.5 and +2.5 indicate significant departure from independence. Adjusted standardised residuals <−8 or >+8 are considered as extreme departures from independence. Positive adjusted residuals in a cell correspond to larger numbers of cases than expected by chance, negative residuals smaller numbers. Class 3 was excluded from the statistical analysis because of the small sample size. (0.03 MB DOC) [file pone.0007107.s005.doc]

|  | **1** | **2** | **3** | **4** | **5** |
| --- | --- | --- | --- | --- | --- |
| **PMA** | ▲ | ▼ |  | ▲ | ► |
| **Flail Arm** | ▲ | ▼▼ |  | ▲ | ► |
| **Flail Leg** | ► | ▼ |  | ▼▼ | ► |
| **ALS** | ▼ | ▲▲ |  | ▼▼ | ▼ |
| **PLS** | ▼ | ▼ |  | ▲▲ | ▲▲ |
| ▲▲ or ▼▼: adjusted standardised residuals > |8| | | | | | |
| ▲ or ▼: adjusted standardised residuals > |2.5| | | | | | |
| ►: adjusted standardised residuals < |2.5| | | | | | |
