# Supplementary figures and images for: Latent Cluster Analysis of ALS Phenotypes Identifies Prognostically Differing Groups
Source: PLoS One. 2009 Sep 22;4(9):e7107. doi: 10.1371/journal.pone.0007107 (PMC2741575; doi:10.1371/journal.pone.0007107)

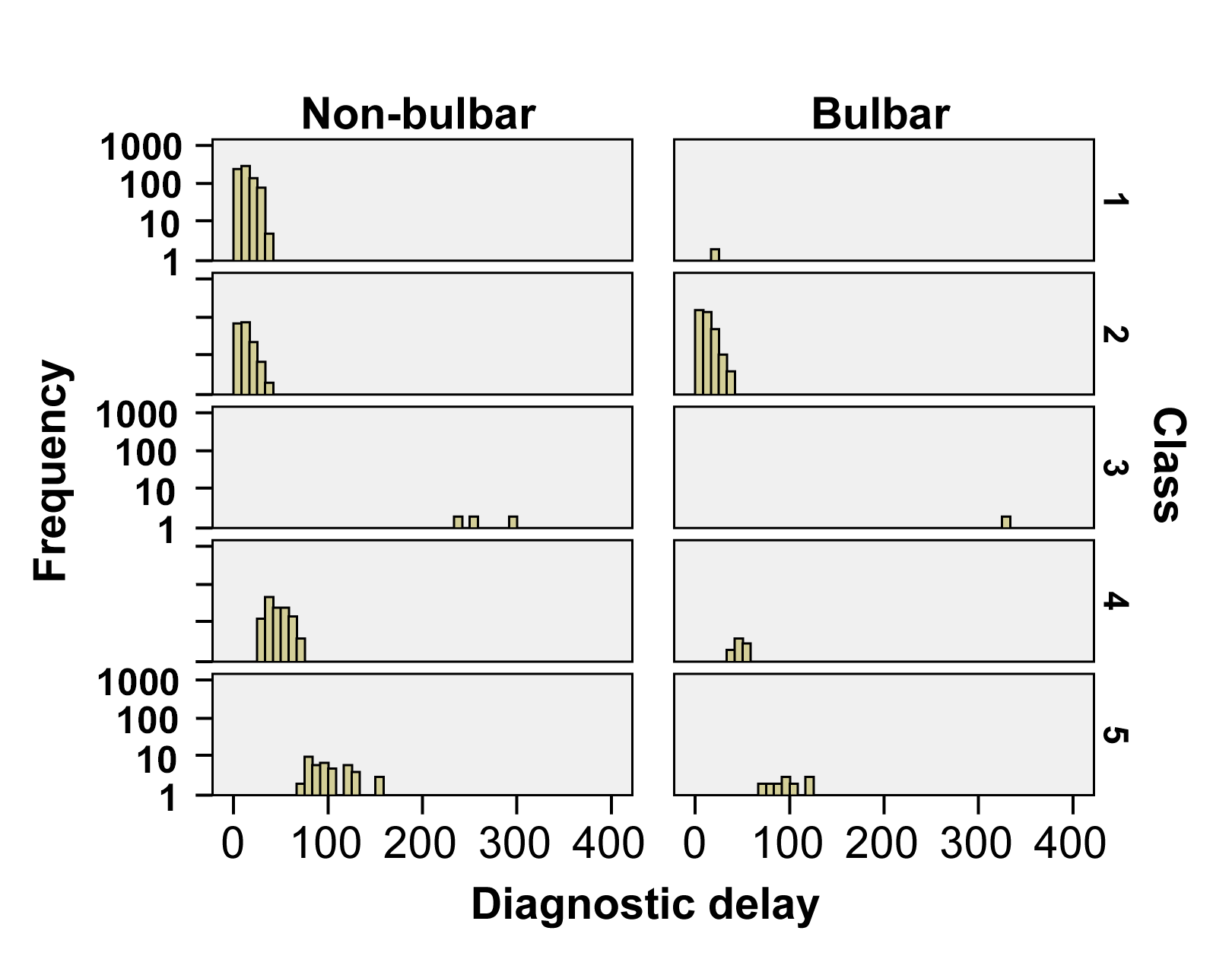

Supplement: Figure S1 — Histogram showing the distribution of diagnostic delay for cases within each class, separately for patients with and without bulbar onset. The frequency (Y-Axis) is shown on a logarithmic scale. (0.27 MB TIF) [file pone.0007107.s006.tif]
